# Crystal structure of AcrB complexed with linezolid at 3.5 Å resolution

**DOI:** 10.1007/s10969-013-9154-x

**Published:** 2013-05-15

**Authors:** Li-Wei Hung, Heung-Bok Kim, Satoshi Murakami, Goutam Gupta, Chang-Yub Kim, Thomas C. Terwilliger

**Affiliations:** 1 Los Alamos National Laboratory, Los Alamos, NM 87545 USA; 2 Life Science Department, School and Graduate School of Bioscience and Biotechnology, Tokyo Institute of Technology, J2-17, 4259 Nagatsuta-cho, Midori-ku, Yokohama, 226-8503 Japan

**Keywords:** Multidrug resistance, AcrB, RND efflux pumps, Linezolid, Membrane protein, Protein–drug complex, X-ray crystal structure

## Abstract

AcrB is an inner membrane resistance-nodulation-cell division efflux pump and is part of the AcrAB–TolC tripartite efflux system. We have determined the crystal structure of AcrB with bound Linezolid at a resolution of 3.5 Å. The structure shows that Linezolid binds to the A385/F386 loops of the symmetric trimer of AcrB. A conformational change of a loop in the bottom of the periplasmic cleft is also observed.

## Introduction

AcrB, the principal multidrug transporter in *E. coli*, crosses the cytoplasmic membrane and acts as a proton/drug antiporter. It is part of the AcrAB–TolC tripartite efflux system consisting of an outer membrane factor, TolC, a periplasmic membrane fusion protein, AcrA, and the inner membrane resistance-nodulation-cell division (RND) efflux pump, AcrB. This integrated three-component molecular complex extrudes a large variety of cytotoxic substances such as antibiotics, organic solvents, dyes, and detergents from the cell directly into the medium, bypassing the periplasm and the outer membrane [1–3]. Over-expression of the tripartite RND efflux systems is thought to be a major factor in multidrug resistance (MDR) in Gram-negative bacteria. Effective control of this and related MDR systems has become an emerging focus for global public health efforts [2, 4].

AcrB is one of the most well-studied RND efflux pumps. Crystal structures of AcrB by itself as well as several drug-bound complexes have been structurally characterized [5–12]. Two types of quaternary arrangement have been observed in structures of AcrB: (1) a symmetric trimer, formed by three identical protomers or protomer–drug complexes; and (2) an asymmetric trimer, consisting of three distinct protomer conformations corresponding to three functional states of the transport cycle: access, binding and extrusion, with drug molecules only bound to the binding protomer. These structures have exemplified several drug-binding modes of AcrB and provided structural insights to the substrate transport mechanism of RND multidrug efflux transporters.

In an effort to explore how AcrB interacts with drugs, we have determined the crystal structure of AcrB complexed with Linezolid, an oxazolidinone-type antibacterial agent that inhibits bacterial protein synthesis by specifically binding to the 50S ribosomal subunit. Linezolid was the first FDA-approved oxazolidinone antibiotic used for the treatment of serious infections caused by Gram-positive bacteria that are resistant to other antibiotics. Therefore, this drug has been called a “reserve antibiotic”, a drug of last resort against potentially intractable infections [13]. Linezolid is a synthetic compound, and is therefore not susceptible to the same mechanisms underlying bacterial resistance against naturally occurring antibiotics. However, it has no clinically significant effect on most Gram-negative bacteria. This is thought to be a result of relatively low intracellular concentration of Linezolid due to efflux [14]. The intracellular concentration of Linezolid could be increased substantially by inhibition of RND-type efflux pumps [14]. *E. coli* with inactivated AcrB has been found to be more susceptible to Linezolid than cells with an intact pump [15]. Further, NMP (1-(1-naphthylmethyl)-piperazine, a putative efflux pump inhibitor) has been shown to reduce the MIC (minimal inhibitory concentration) of Linezolid by fourfold for *E. coli*, *Citrobacter freundii*, *Enterobacter aerogenes* and *Acinetobacter baumannii* [16, 17]. Although these data suggest that Linezolid can be extruded by efflux pumps, there is no direct evidence yet to support this hypothesis. Here, we report the crystal structure of AcrB and Linezolid complex, in which AcrB indeed binds Linezolid in the same fashion as several other antibiotics that are extruded by efflux pumps.

## Materials and methods

### Cloning, overexpression, and purification

Wild-type AcrB with a C-terminal polyhistidine tag was prepared as described previously [7]. Briefly, AcrB was overproduced in *E. coli* JM109 with a histidine-tagged AcrB-overexpersion plasmid pAcBH. The cells were disrupted with Microfluidizer (Microfluidics Corp.) and the membrane fractions were collected washed using several ultracentrifugation steps at 150,000*g* for 90 min. Purified membranes were solubilized in buffer containing 50 mM Tris–HCl, pH 7.0, 10 % glycerol in 2 % *n*-dodecyl-β-d-maltoside (DDM) (Anatrace). Lipids and debris were removed by ultracentrifugation at 170,000*g* for 60 min. Extracted histidine-tagged AcrB was purified with metal affinity column chromatography equilibrated with buffer (20 mM Tris–HCl, pH 7.5, 0.3 M NaCl, 10 % glycerol and 0.2 % DDM). The column was washed stepwise using 25 and 100 mM imidazole added to the above buffer. Purified AcrB was eluted with 300 mM imidazole. Imidazole was then removed by three concentration-dilution steps using an ultrafiltration membrane. Proteins were concentrated to 28 mg/mL in 20 mM sodium phosphate (pH 6.2), 10 % (v/v) glycerol and 0.2 % (w/v) DDM and were frozen in liquid nitrogen.

### Crystallization and data collection

AcrB was crystallized using the sitting-drop vapor diffusion method with 0.1 M NaCl, Na-phosphate pH 6.2, and 8 % PEG 4000 as crystallization reagents. Crystals of the AcrB–Linezolid complex were obtained by soaking the AcrB crystals in a solution containing Linezolid prior to data collection. Linezolid stock solution (30 mM) was prepared with water, and 6 mM Linezolid was added to a cryosolvent containing the crystallization reagents plus 25 % glycerol. Apo-AcrB crystals were transferred to the cryosolvent and incubated at 21 °C for 10 min before flash cooling in liquid nitrogen. X-ray diffraction data were collected at 100 K on the 5.0.2 beam line at the Advanced Light Source at the Lawrence Berkeley National Laboratory with X-rays at a wavelength of 1 Å. The crystal diffracted better than 3.3 Å initially and decayed during data collection, leading to a useful resolution of about 3.5 Å by the end of data collection. The diffraction data were processed with the HKL2000 program suite [18]. The AcrB–Linezolid complex belongs to the space group *R32* with cell parameters *a* = *b* = 144.7 Å, *c* = 519.4 Å. The solvent content is 74 % assuming 1 molecule in the asymmetric unit.

### Structure determination and refinement

**Fig. 1:**
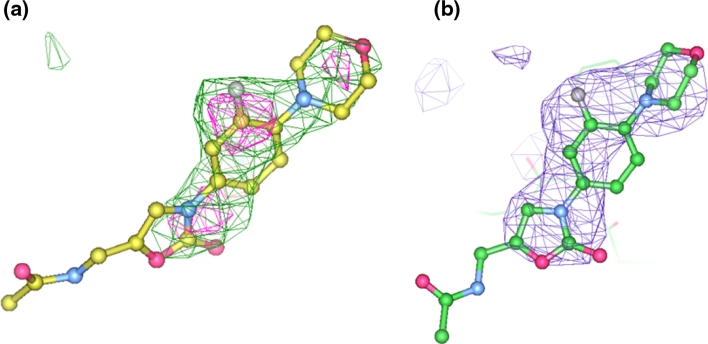
**a** Unbiased Fo–Fc difference Fourier map contoured at 3σ (*green*) and 3.6σ (*magenta*) with a Linezolid molecule overlaid onto the density. No Linezolid had been modeled and refined at this stage. **b** Final refined 2Fo–Fc map contoured at 1σ

The structure determination and refinement was conducted with the *Phenix* program suite [19]. The apo-AcrB crystal structure (1IWG, [7]), obtained using conditions similar to those used here and having the same space group and very similar cell parameters (*a* = *b* = 144.5 Å, *c* = 519.2 Å) was used as starting model for refinement using *phenix.refine* [20]. The asymmetric unit of the crystal contains one chain of AcrB. One round of rigid-body refinement followed by B-factor refinement yielded an R-factor of 30.9 %. The refinement was continued with several cycles of positional, B-factor, and TLS refinement, and revealed clear difference density (Fig. 1a, green, contoured at 3σ) near residue F386. Increasing the contour level of the Fo–Fc map to 3.6 σ revealed 3 separate blobs (Fig. 1a, magenta) suggesting that the density consisted of 3 electron-dense substructures, consistent with the three separate electron-dense parts of Linezolid. A Linezolid was therefore modelled into this density. In addition, residues 860–864 were rebuilt into the electron density map as they were not modelled in the starting model. The structure was manually inspected and minor changes were made in Coot [21], followed by refinement until convergence. The final refined structure has an R-factor of 25.1 % and a free-R factor of 30.4 % for data between 50 and 3.5 Å. These are similar to the R-factors for the earlier AcrB structure (29.0/35.5 %). As a comparison, the R- and free R-factors of AcrB–Linezolid complex without TLS refinement were 29.8 and 33.7 %, respectively. Detailed statistics are shown in Table 1. The final refined Linezolid model and density are shown in Fig. 1b.

**Table 1:** X-ray data and refinement statistics

*Crystal parameters*	
Space group	R 32
Cell dimensions	
a, b, c (Å)	144.7, 144.7, 519.4
α, β, γ (°)	90, 90, 120
Matthews coefficient (Å^3^/Da)	4.8
Solvent content (%)	74.2
*Data collection* ^a^	
Wavelength (Å)	1.0
Resolution (Å)	46.6–3.5 (3.56–3.50)
R_merge_ (%)	6.1 (78.8)
No. of unique reflections	25,688
No. of reflections in R_free_ set	1,788
Mean redundancy	5.8 (5.9)
Overall completeness (%)	99.8 (100.0)
Mean I/σ	28.0 (2.2)
*Refinement residuals* ^b^	
R_free_ (%)	30.36 (44.0)
R_work_ (%)	25.13 (35.9)
Completeness (%)	95.3 (56.1)
*Model quality*	
RMSD bond lengths (Å)	0.003
RMSD bond angles (°)	0.767
MolProbity Ramachandran distribution	
Most favored (%)	88.0
Allowed (%)	9.7
Disallowed (%)	2.3
Mean main chain B-factor (Å^2^)	130
Mean overall B-factor (Å^2^)	133
Mean solvent B-factor (Å^2^)	N/A
*Model contents*	
Protomers in ASU	1
Protein residues	A (7-498, 513-864, 869-1,036)
Ligands	1 Linezolid
No. of protein atoms	7,676
No. of ligand atoms	24
No. of water molecules	0
PDB accession code	4K7Q

Standard definitions were used for all parameters. Entries in parentheses report data from the limiting resolution shell. Data collection and refinement statistics come from *HKL2000* and *PHENIX*, respectively. The abbreviations RMSD and ASU stand for root-mean-square deviation and asymmetric unit, respectively

^a^All observations with I ≥ −3σ_I_ were included in calculating data-quality statistics. Rmerge = Σhkl Σi |Ii(hkl) − <I(hkl)>|/Σhkl Σi Ii(hkl) where Ii(hkl) is the intensity of the ith observation and <I(hkl)> is the mean intensity of the reflections

^b^The crystallographic R factor R = Σhkl | |Fobs| − |Fcal| |/Σhkl |Fobs|; Rfree = Σhkl | |Fobs| − |Fcal| |/Σhkl |Fobs| where all reflections belong to a test set of randomly selected data

### Structure validation and deposit

The quality of the final structure was assessed using *MolProbity* [22] and *phenix.refine*. The atomic coordinates and structure factors are available in the Protein Data Bank under accession code 4K7Q.

## Results and discussion

### Overall structure of AcrB–linezolid complex

**Fig. 2:**
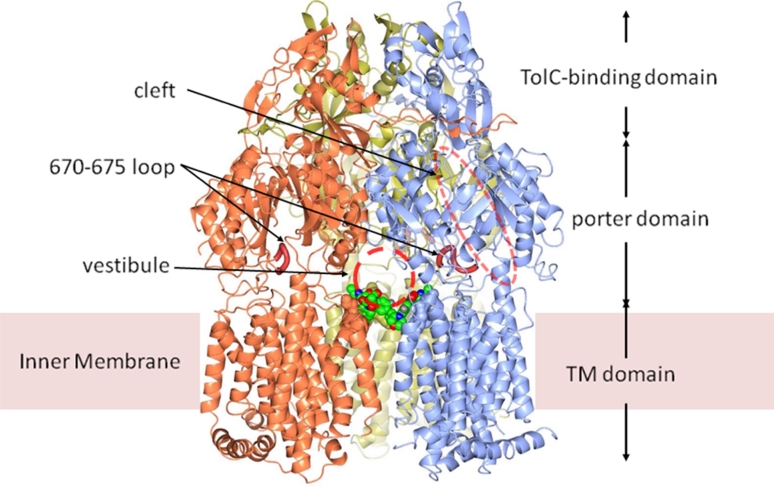
Schematic diagram of AcrB–Linezolid complex. The Linezolid molecules are in colored space-filling model, viewable through one of the vestibule tunnels. The symmetric AcrB protomers are in *ice-blue*, *coral*, and *gold ribbons*. The 670–675 loops are shown in crimson tubes as depicted. Other components of the AcrB trimer described in the text are also labelled. This figure was created with CCP4 mg [27]

Figure 2 shows a ribbon diagram of the complex structure of AcrB–Linezolid with Linezolid molecules shown in space-filling models. The AcrB crystal used for Linezolid soaking was in the *R32* space group in which symmetric trimers form in the unit cell. Each AcrB monomer contains a transmembrane (TM) domain consisting of 12 TM helices, and two periplasmic domains, the porter domain, and the TolC-binding domain [7]. The AcrB monomers form a trimer which appears to be stabilized by the inter-monomer locking loops protruding into the adjacent AcrB monomer in the TolC binding domain. The interlocked TolC-binding domains form a funnel-like structure at the top and a connected tunnel at the center. The tunnel leads through the porter domain down to the large central cavity formed by the TM domains of the three protomers. The central cavity is connected also to the periplasm through three vestibules located at subunit interfaces. These vestibules have been shown to play important roles in substrate capture and transport [23]. A cleft at the periplasmic periphery of the porter domain has been suggested to accommodate AcrA [7], and has been shown to be crucial for substrate binding and transport of AcrB [24, 25].

**Fig. 3:**
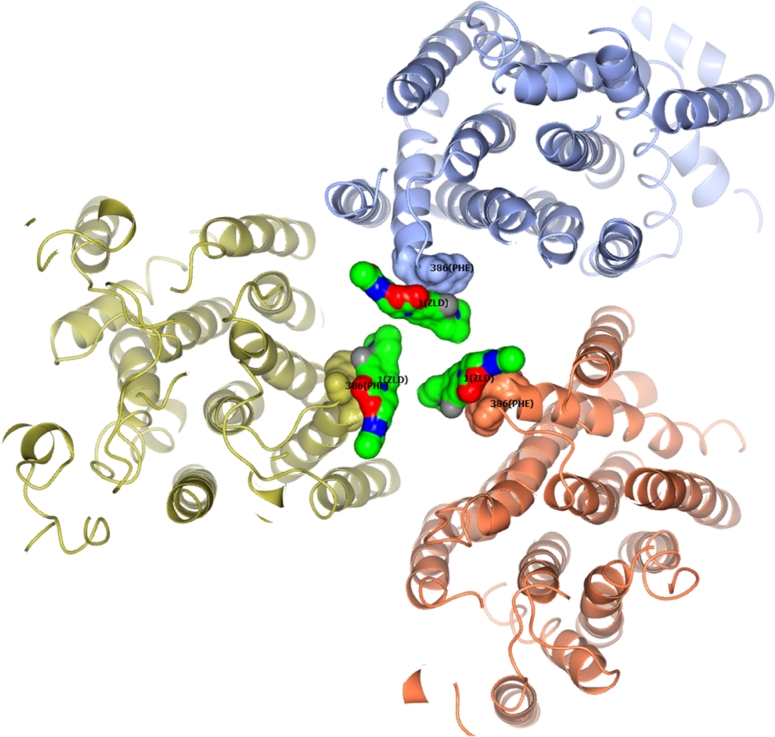
Molecular contacts of AcrB trimer and Linezolid. The view is centered on the threefold axis on the periplasmic side looking down the inner membrane. The helixes shown are TMs of AcrB. Residues A385 and F386 of AcrB as well as Linezolid molecules are shown in colored surface models. Positions of F386 and Linezolid (ZLD) are labeled. The rest of the AcrB are in *ribbons* representation. The Linezolid molecules are in *green*, *blue*, *red*, colors indicating positions of C, N, O atoms, respectively. The colors are not indications of charges or potentials. This figure was prepared with CCP4 mg

Each protomer of the symmetric AcrB trimer binds one Linezolid molecule on the wall of the upper portion of the central cavity near residues A385 and F386. The 3 rings of the Linezolid molecule lie approximately parallel to the F386 binding loop, allowing maximum hydrophobic contact between the drug and AcrB (Fig. 3). The binding interface buries approximately 140 Å^2^ surface of Linezolid (calculated with *areaimol*, [26]). Almost the whole Linezolid except for its acetamide tail participates in this intermolecular interaction dominated by hydrophobic stacking. Several other drugs including ethidium [12], nafcillin [11] and ampicillin [10] also have been found to bind to this location on the symmetric AcrB trimers.

### Comparison with apo-AcrB structure

The overall structure of AcrB–Linezolid complex was very similar to that of AcrB by itself [7]. There was a significant local conformational difference at residues 670–675. When superposed together, the two structures has a root-mean-square Cα coordinate deviation of 0.4 Å while these residues, on the surface of the protein but not near crystal contacts, differ by up to 4 Å in backbone position. These residues reside in a loop lining the bottom of the cleft in the porter domain thought to be important for substrate transport and AcrA binding. It is possible that the change in position of this loop between unbound and Linezolid-bound states may reflect a functionally important state of AcrB, though we cannot rule out the possibility that the shift in the 670–675 loop was due to slight differences between crystals used for structure determination. We are in the process of obtaining higher resolution data of the AcrB–Linezolid complex as well as AcrB by itself under identical crystallization and data collection conditions.

## Summary

The crystal structure of an AcrB–Linezolid complex has been determined at a resolution of 3.5 Å. The structure shows that one Linezolid binds to the A385/F386 loop of each protomer in the symmetric trimer. This loop has previously been shown to interact with Ethidium, Nafcillin, and Ampicillin. A conformational change is also found in a loop at the bottom of the periplasmic cleft thought to be important for AcrA binding and drug transport.

## Abbreviations

AcrA: Acriflavine resistance protein A
AcrB: Acriflavine resistance protein B
ASU: Asymmetric unit
FDA: US food and drug administration
Linezolid: (*S*)-*N*-({3-[3-fluoro-4-(morpholin-4-yl)phenyl]-2-oxo-1,3-oxazolidin-5-yl}methyl)acetamide
MIC: Minimal inhibitory concentration
MDR: Multidrug resistance
NMP: 1-(1-Naphthylmethyl)-piperazine
PEG: Polyethylene glycol
PDB: Protein data bank
RND: Resistance nodulation division
TM: Transmembrane
